# Body size and lower limb posture during walking in humans

**DOI:** 10.1371/journal.pone.0172112

**Published:** 2017-02-13

**Authors:** Martin Hora, Libor Soumar, Herman Pontzer, Vladimír Sládek

**Affiliations:** 1 Department of Anthropology and Human Genetics, Faculty of Science, Charles University, Prague, Czech Republic; 2 CASRI - Sports Research Institute of Czech Armed Forces, Prague, Czech Republic; 3 Department of Anthropology, Hunter College, New York, New York, United States of America; Universite de Nantes, FRANCE

## Abstract

We test whether locomotor posture is associated with body mass and lower limb length in humans and explore how body size and posture affect net joint moments during walking. We acquired gait data for 24 females and 25 males using a three-dimensional motion capture system and pressure-measuring insoles. We employed the general linear model and commonality analysis to assess the independent effect of body mass and lower limb length on flexion angles at the hip, knee, and ankle while controlling for sex and velocity. In addition, we used inverse dynamics to model the effect of size and posture on net joint moments. At early stance, body mass has a negative effect on knee flexion (p < 0.01), whereas lower limb length has a negative effect on hip flexion (p < 0.05). Body mass uniquely explains 15.8% of the variance in knee flexion, whereas lower limb length uniquely explains 5.4% of the variance in hip flexion. Both of the detected relationships between body size and posture are consistent with the moment moderating postural adjustments predicted by our model. At late stance, no significant relationship between body size and posture was detected. Humans of greater body size reduce the flexion of the hip and knee at early stance, which results in the moderation of net moments at these joints.

## Introduction

The loading of the musculoskeletal system during locomotion and the metabolic cost of locomotion are primarily determined by muscle force production required to support, propel, and control the balance of the body and move the limbs [1–11]. The force that has to be generated by muscles during terrestrial locomotion is primarily affected by morphological and gait characteristics, terrain, and surface properties [4,9,12–15]. Body mass and lower limb length were shown to be positively related to muscle force requirements. With greater body mass, muscles have to produce greater forces to support the body weight [3,16], whereas longer lower limb prolongs the moment arms of the joint reaction forces and increases the moment of inertia of the lower limb segments [17]. The reported negative effect of lower limb length on metabolic cost of locomotion [18–20] is likely a consequence of the covariation between lower limb length and parameters such as step length, stance time [3,9,21], and muscle moment arm lengths [22]. Muscle moment arm lengths are negatively related to muscle force production and consequently to locomotor cost (but see [23]) and bone loading [2,24]. The locomotor muscle force demands are significantly affected also by gait characteristics such as stance time [3], posture (i.e., the position of body segments relative to each other and to the ground) [2], and generally by velocity which affects both the stance time and posture [3,7,25]. Stance time has been shown to be inversely related to muscle force demands [3,8] as was more extended posture due to its effect on moment arms of the joint reaction forces [2]. Prolonged stance time and adoption of more extended posture could thus be viable mechanisms for moderation of increased muscle force demands of animals with greater body mass and/or lower limb length [2,3]. Although, such moderating relationship between body size and posture in particular have been demonstrated at an interspecies level [2,26], the evidence for its presence within species is contradictory, which may be partly due to insufficient control of other posture-affecting factors.

Among a phylogenetically diverse sample of mammals ranging from rodents to ungulates, species of greater body size keep their limbs more extended during the stance phase of locomotion [2,27]. Contradictory results come from studies of taxonomically narrow and phylogenetically close mammal groups, however, such as within families. The significant relationship between body size and posture was detected among terrestrial monkeys [28,29]. Particularly, Polk [28] reported that larger *Cercopithecinae* monkeys (*Chlorocebus aethiops*, *Erythrocebus patas*, and *Papio anubis*) had more extended elbow and shoulder joints at mid stance during walking, whereas Patel et al. [29] reported that older, heavier baboons (*Papio hamadryas ursinus*) tend to walk with more extended knees. In contrast, no relationship between body size and posture was detected among cats (*Felidae*) [30] or elephants (*Elephantidae*) [31], despite great variation in body size in both samples (46-fold and seven-fold body size range, respectively).

The effect of body size on human locomotor mechanics is understudied and unclear. Few studies have detected a relationship between body size and locomotor posture, but these studies were either not supported by others or did not control for other factors affecting posture. In humans, more extended lower limbs during the stance phase of walking were associated with both greater body mass [32–34] and lower limb length [35]. However, the effect of body mass was studied either using small sample size [32] or through the comparison of lean and obese subjects only [33,34], while gait may also be altered by factors other than body mass (e.g., pain or mass distribution). Moreover, other studies did not detect differences in posture between lean and obese humans [36–39]. On the other hand, the effect of lower limb length was studied without controlling for body mass [35], which is usually correlated with lower limb length. Thus, it cannot be ruled out that part of the detected lower limb length effect should actually be ascribed to body mass.

Sex could be a confounding factor in studies of human locomotor posture, as human males and females, who differ significantly in body size, do not differ in lower limb posture during the stance phase of walking [40–42]. Moreover, some studies have even reported that males, despite their greater body size, keep their limbs more flexed than females do at least at some joints during the stance phase of walking [43–46]. Despite these contradictory findings, the effect of sex was not considered in previous studies of the body size-posture relationship in humans.

Velocity is another factor affecting walking posture in humans. Generally, velocity has a positive effect on flexion at the hip and knee and plantarflexion at the ankle during the stance [44,47–53]. As such, velocity must be controlled for when assessing the relationship between body size and posture. In previous studies, posture was usually analyzed at a self-selected velocity (e.g., [33]) or at a standard velocity if samples of equal average stature were compared (e.g., [36]) to account for the velocity effect.

The postural adjustments associated with greater body size were linked to changes in net joint moments in previous studies. The estimation of the force generated by particular muscles through forward dynamics simulation requires complex musculoskeletal modeling approach and estimation of several parameters such as muscle fiber length, tendon rest length, and force-length properties of tendons and ligaments which are impossible to validate in living subjects [10] yet have substantial effect on the muscle force estimates [54]. On the other hand, the net joint moment that reflects the net muscle moment exerted about a particular joint by all the agonist and antagonist muscles, can be estimated relatively easily through inverse dynamics [55]. It is thus no surprise that the net joint moments were used as a proxy for musculoskeletal loading in previous studies (e.g., [2,56,57]; but see limitations of this approach in [55,58] and below). Notably, inverse dynamics studies indicate that the lower peak knee flexion angle in early stance is associated with a decrease of peak knee flexion moment in obese adults and children [33,34]. In addition, Gruss [35] characterized the more extended knee position at late stance in longer limbed individuals as a compensatory mechanism that moderates the knee flexion moment. Nevertheless, we suggested elsewhere [32] that the net knee flexion moment at late stance is relatively low and even absent in some individuals. Therefore, it is not so evident that the relationship between knee angle and lower limb length detected by Gruss [35] (but not by others [32]) represents a knee moment moderation mechanism.

Although the inductive approach used in these studies identifies interesting statistical relationships, further insight into their function could be provided by a modeling approach. An appropriate biomechanical model would allow independent manipulation of the parameters such as body mass, lower limb length, and posture to identify their particular effect on the net joint moments. Various models were employed in studies of biomechanics of human locomotion from relatively simple models allowing analyses of basic gait parameters and general energetics of the gait [8,59,60] to very complex musculoskeletal models in which function of particular muscles is assessed [10,61,62]. In the present study, we will use the link-segment model [55,63] along with the divergent point (DP) model of Gruben and Boehms [64]. This modelling approach is complex enough to provide estimates of net joint moments while allowing manipulation of the anthropometric and kinematic parameters, yet it is sufficiently simple for easy result interpretation. This approach enables us to examine size effects in human gait and relate these findings to comparative analyses of animal locomotion and scaling [2,27–31].

**Fig 1:**
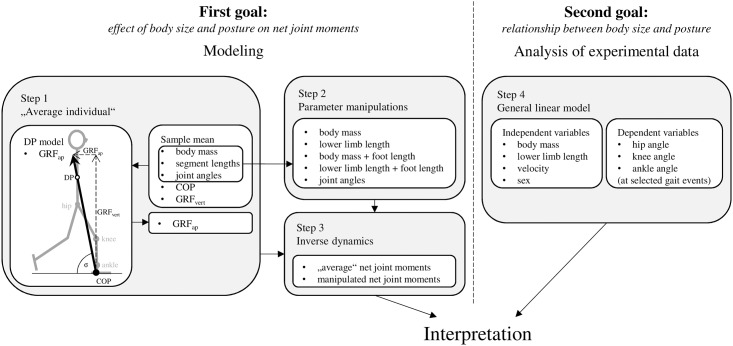
Schematic diagram of the approaches used to accomplish the goals of this study.

In the present study, we test the prediction that humans adjust their posture during walking to minimize the size-related increase of net moments acting on their lower limb joints (Fig 1). The first goal is to identify postural adjustments that moderate the net joint moments in human walking. This goal is accomplished by modeling the effect of body size and posture on net joint moments (Steps 1–3). The second goal is to experimentally test the impact of body mass and lower limb length on lower limb posture during walking in a non-obese human sample while controlling for other posture-affecting factors (Step 4). Based on previous studies, we expect that both body mass and lower limb length will be associated with walking posture in humans. We further expect that size-related postural adjustments take place at those periods of stance at which net moments act to flex the hip and knee and dorsiflex the ankle. The general linear model is used to assess the independent effect of body mass and lower limb length on the posture of walking humans while controlling for sex and velocity. Finally, we use the results of our modeling to interpret the results of the analysis of the experimental data. Particularly, we compare whether the body size-related postural adjustments identified experimentally correspond with moment moderation adjustments predicted by our model.

## Materials and methods

### Sample

This study was approved by Institutional Review Board of Charles University, Faculty of Science, approval number 2011/2. Each participant signed a consent form which was also approved by the Institutional Review Board of Charles University, Faculty of Science. Forty-nine volunteers, 25 males and 24 females, participated in this study. Participants were between 19 and 38 years of age, non-obese (body mass index < 30 kg m^−2^), and had no history of lower limb or spine injuries or illnesses. Participants were selected with the aim to maximize variation in body size.

### Anthropometry

Two anthropometric parameters representing body size were used in the present study: body mass and lower limb length. Body mass was measured with a digital weighing scale just prior to the gait data collection. Lower limb length was determined as the sum of thigh length and shank length, both measured in Visual3D software (C-Motion, Germantown, MD, USA) using 3D spatial data acquired via motion capture system (Qualisys, Gothenburg, Sweden) during the standing trial. Thigh length was defined as the distance between the hip center of rotation (approximately the center of the femoral head; see below) and the knee center of rotation (midpoint between the lateral and medial epicondylus). Shank length was defined as the distance between the knee center of rotation and the ankle center of rotation (midpoint between the distal apexes of the lateral and medial malleolus). These measurements differ from standard osteometrics, as thigh length is less than femoral bicondylar length and shank length is greater than tibial maximum length. The sum of these measurements, however, reflects the lower limb length as determined by standard osteometrics better than other somatometrics whereby the lower limb length is usually defined as a distance between the greater trochanter and the most medially prominent point on the malleolus (thus shorter than the sum of the corresponding bone lengths). In addition, we measured foot length (M 58) [65] using osteometric board. Sample statistics of anthropometric parameters are given in Table 1.

**Table 1:** Sample statistics of anthropometric parameters.

Sex		Stature (mm)	Body mass (kg)	Body mass index (kg m^−2^)	Lower limb length (mm)	Shank length (mm)	Thigh length (mm)	Foot length (mm)
Males (n = 25)	Mean (SD)	1821 (80.1)	75.5 (13.05)	22.7 (3.14)	860 (52.0)	433 (27.9)	427 (26.7)	271 (14.7)
	Min–Max	1658–1986	53.2–97.1	16.6–29.1	746–976	373–488	373–491	229–293
Females (n = 24)	Mean (SD)	1678 (91.0)	61.3 (12.46)	21.6 (3.32)	788 (52.4)	396 (27.6)	391 (29.1)	248 (13.6)
	Min–Max	1533–1863	36.3–77.8	15.4–27.5	703–883	345–441	337–441	227–285
Pooled (n = 49)	Mean (SD)	1751 (111.6)	68.5 (14.51)	22.2 (3.24)	825 (63.2)	415 (33.0)	410 (32.9)	260 (18.1)

### Gait analyses

Gait data were collected in the biomechanical laboratory of CASRI—Sports Research Institute of the Czech Armed Forces. Participants walked on a level treadmill (h/p/cosmos, Nussdorf-Traunstein, Germany) at their preferred velocity (mean ± standard deviation: 4.99 ± 0.53 km h^−1^; range: 3.5–6.0 km h^−1^) while their kinematic and kinetic data were recorded. Prior to data collection, participants acclimatized to treadmill locomotion for approximately 25 minutes. Their preferred velocity was used with the aim of standardizing the effect of velocity on locomotor posture. The preferred walking velocity of each participant was established at the end of the acclimatization session by increasing the speed in 0.1 km h^−1^ increments from a relatively slow speed until the participant reported to walk at his or her preferred velocity. The velocity was then increased by 1.5 km h^−1^ and thereafter decremented by 0.1 km h^−1^ until the preferred velocity was re-established [66]. The mean of the two established velocities was then taken as the individual’s preferred velocity. Participants rested for at least 30 minutes between establishing the preferred walking speed and gait data collection. Participants wore their own sports shorts and T-shirts and were provided with uniform neoprene shoes (Hiko Softy, Prague, Czech Republic) with a thin sole to imitate barefoot walking. Thin sole was used with the aim to control for the presumed effect of shoes on gait parameters [67–69] and specially to allow the application of the results also in studies of past human populations [70]. Kinematic data were collected with a 10-camera three-dimensional motion capture system (Qualisys, Gothenburg, Sweden) at a 100 Hz frequency. Vertical ground reaction force and center of pressure (COP) data were collected with pressure-measuring insoles (Pedar, Novel, Munich, Germany) at a 100 Hz frequency. Marker trajectories, vertical GRF and COP path were synchronously recorded for 10 seconds. The data from pressure-measuring insoles were used only for determination of the timing of the vertical GRF peaks. In addition, the mean vertical GRF was used as an input parameter in our model of an average walking human (see below).

**Fig 2:**
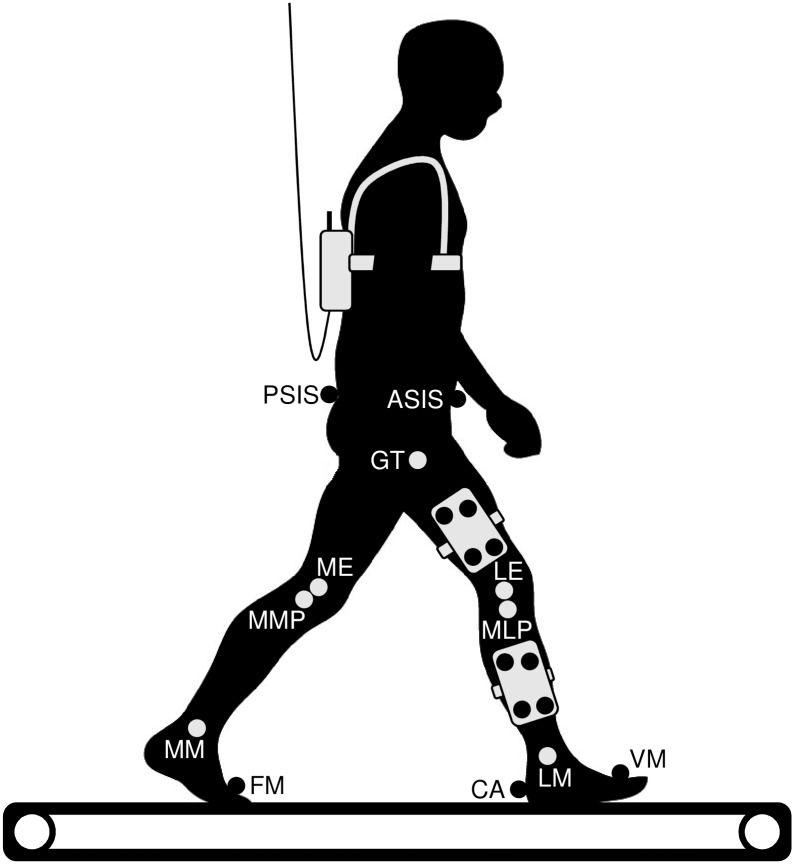
Diagram of the experimental setup showing location of the tracking markers (black circles), recorded bony landmarks (light grey circles), and Pedar unit (at the back of the subject). Note that all the markers and landmarks were recorded bilaterally. ASIS, anterior superior iliac spine; PSIS, posterior superior iliac spine; CA, tuber calcanei; FM, head of the first metatarsal bone; VM, head of the fifth metatarsal bone; GT, greater trochanter; ME, medial epicondyle; LE, lateral epicondyle; MMP, most medial point of the ridge of the medial tibial plateau; MLP, most lateral point of the ridge of the lateral tibial plateau; MM, distal apex of the medial malleolus; LM, distal apex of the lateral malleolus.

A modified calibrated anatomical systems technique (CAST) [71] was used to track the lower limbs’ kinematics (Fig 2). Our model consists of four segments: pelvis, thigh, shank, and foot. The pelvis was tracked by markers at the anterior superior iliac spines and posterior superior iliac spines. The thigh and shank were each tracked by four markers attached to a rigid plate. The foot was tracked by markers at the tuber calcanei and heads of the first and fifth metatarsal bones. Additionally, the locations of seven bony landmarks (greater trochanter, medial epicondyle, lateral epicondyle, the most medial point of the ridge of the medial tibial plateau, the most lateral point of the ridge of the lateral tibial plateau, the distal apex of the medial malleolus, and the distal apex of the lateral malleolus) per limb in relation to marker clusters were found by manual palpation and recorded using a digitizing pointer. Our modification from the CAST technique consists of the replacement of the marker on the head of the fibula by a marker on the ridge of the lateral tibial plateau and of the abandonment of the marker on the second metatarsal head. The joint coordinate system was defined following Grood and Suntay [72] and International Society of Biomechanics recommendations [73]. The hip center of rotation was estimated by a functional approach developed by Schwartz et al. [74], the protocol of thigh movement (10 cycles of limited thig flexion-extension, abduction-adduction, and circumduction) followed Begon et al. [75].

**Fig 3:**
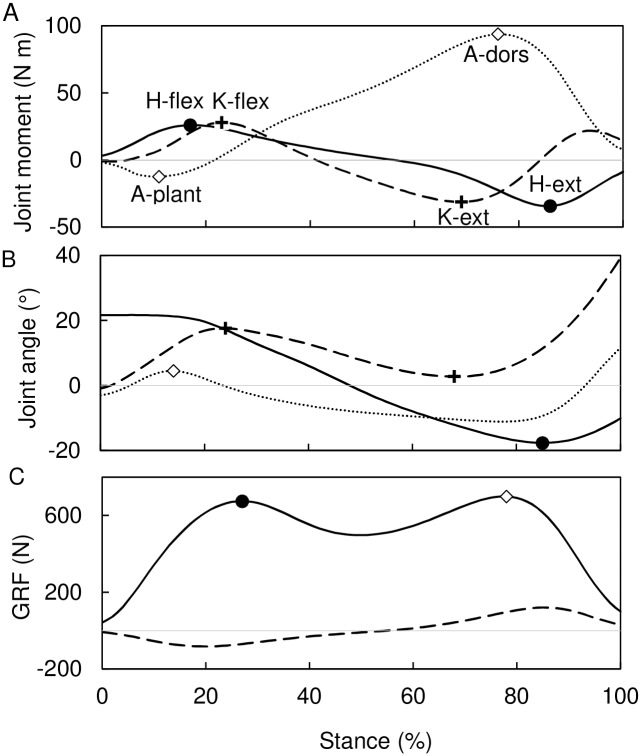
Determination of gait events used to track the peak net joint moments. (A) Illustrative joint moments at the hip (solid line), knee (dashed line), and ankle (dotted line) with peak moments indicated. (B) Sample mean angular displacement of the hip (solid line), knee (dashed line), and ankle (dotted line) with gait events used to track peak moments indicated (open diamond, A-plant; crosses, K-flex and K-ext; filled circle, H-ext). (C) Sample mean vertical ground reaction force (solid line) and estimated antero-posterior ground reaction force (dashed line) with gait events used to track peak moments indicated (filled circle, H-flex; open diamond, A-dors). See text for definitions of peak moments and gait events.

The raw kinematic, vertical ground reaction force, and COP data were filtered using a fourth-order low-pass Butterworth filter with a 6 Hz cut-off frequency [55] in Visual3D software. The stance phase of each stride was delimited using a velocity-based detection algorithm [76] verified by visual inspection. Averages of three to nine strides per individual were used in further analyses. Joint flexion angles at the hip, knee, and ankle were used in analyses. The coordinate system of the pelvis segment was normalized to standing posture prior to joint angles computation. We avoided any other segment or joint angle adjustments to standing posture. The joint flexion angles used in this study are thus those between mechanical axes of segments. Joint flexion angles of the dominant lower limb were processed in the ensuing analyses. The dominant lower limb was defined as that used to manipulate an object or to lead out as in jumping [77,78] and was identified by questionnaire inquiring as to the preferred lower limb in different activities (kicking a ball, hopping on one foot, stepping on a chair, and stamping on an object). Mean joint flexion angles and vertical ground reaction force of the pooled-sex sample during stance phase are given in Fig 3.

### Modeling of body size and posture effect on joint moments

To assess how body size affects net joint moments (our first goal) and anticipate the timing and magnitude of the expected size-related postural adjustment, we modeled the effect of body size and postural changes on net joint moments by manipulating body size and posture in an “average individual” (Fig 1).

#### Step 1: “Average individual”

First, an average walking individual was constructed using the mean segment dimensions, vertical GRF, COP path, and kinematics of our human sample (Fig 3, Table 1, S1 File). The anteroposterior GRF (GRF_ap_) was calculated from the vertical GRF (GRF_vert_) estimated with software provided by Novel as follows:

$$GRF_{ap}=\frac{GRF_{vert}}{\text{tan}\sigma }$$ (1)

where σ is the angle between the GRF vector and the horizontal. The angle σ was estimated using the DP model of Gruben and Boehm [64], i.e., the orientation of the GRF vector was defined by the location of the instantaneous COP and the divergent point (DP) of the GRF located 54% of the center of mass of the body (COM_body_) height vertically above the hip joint, which is the mean DP location for a sample of walking adult humans reported by Gruben and Boehm [64]. As documented by previous experimental studies [64,79] this approach should provide appropriate estimates of the GRF vector orientation and GRF magnitude throughout the stance (see also our assessment of the DP model accuracy below). The height of the COM_body_ used in localization of the DP was calculated from seven body segments (feet, shanks, thighs, and head-arms-trunk segment) at standing posture following Winter [55].

#### Step 2: Parameter manipulation

Second, we manipulated body mass, lower limb length (i.e., the sum of thigh length and shank length), and flexion angles at the hip, knee, and ankle of our average individual by adding and subtracting two standard deviations (SD) of our sample (see Table 1 for SD of anatomical parameters; mean flexion angle SD across the joints and stance phase = 4°) and calculated the resulting net joint moments (Fig 1). The addition and subtraction of two SDs was done to get simulations representing the range of a normal human variation in the manipulated parameters. Each parameter was manipulated independently while all other parameters, including stance time and relative position of the segments´ COM, were held constant unless specified below.

Body mass manipulation was allowed to affect the GRF_vert_:

$$GRF_{vert}=BM\times normGRF_{vert}$$ (2)

where BM is the body mass after manipulation and normGRF_vert_ (N kg^−1^) is the vertical GRF of our average individual normalized to his body mass. In addition, body mass affected in a direct proportion also the mass of the lower limb segments.

Lower limb length manipulation was accomplished by simultaneous proportional change of thigh length and shank length so that the ratio of shank to thigh length remained constant. This solution is supported by the research of Holliday [80] who showed that the ratio of shank to thigh length explains only 4% of the variance in lower limb length. Lower limb length manipulation caused no changes in ankle height or foot length.

Nevertheless, foot length is positively correlated with both body mass (r^2^ = 0.603) and lower limb length (r^2^ = 0.639) in our sample. Moreover, each of these size parameters have similar, significant effect on foot length even when the other size parameter is controlled for by multiple regression analysis as revealed by standardized coefficients (β_body mass_ = 0.421; β_lower limb length_ = 0.499). Thus, to accommodate this relationship between body size and foot length, we additionally manipulated body mass and lower limb length together with foot length. The foot length was manipulated using regression slopes from the multiple regression analysis (b_body mass_ = 0.524 mm kg –1; b_lower limb length_ = 0.143 mm mm^–1^). The progression of the COP relatively to foot length was kept constant, thus at any percentage of stance phase the COP was located at the same relative distance from the posterior end of the sole in the models with manipulated foot length and in the model of an average individual.

**Fig 4:**
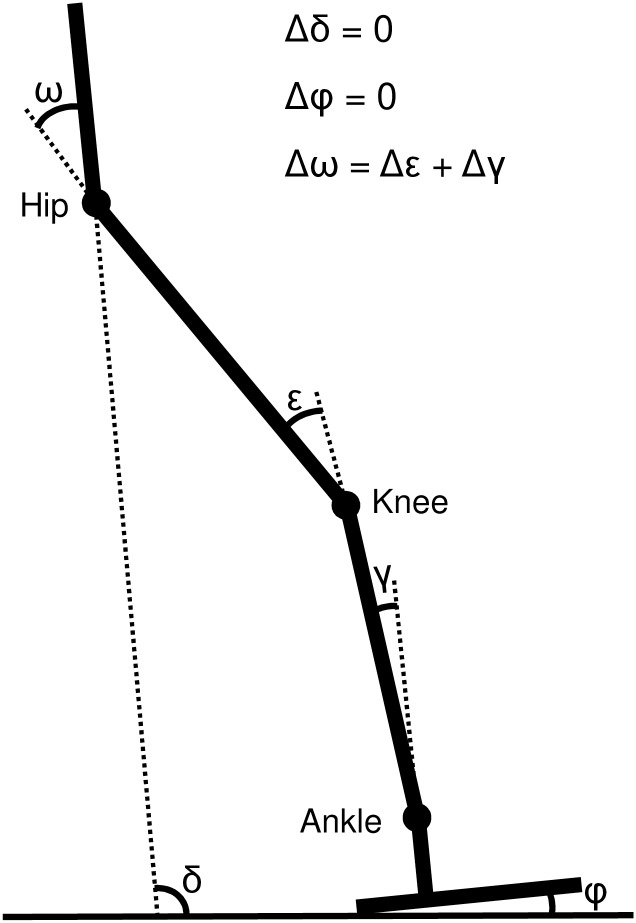
The pelvic tilt (δ) and foot angle (φ) are not affected by postural changes of the hip (ω), knee (ε), and ankle (γ) in the present model.

**Fig 5:**
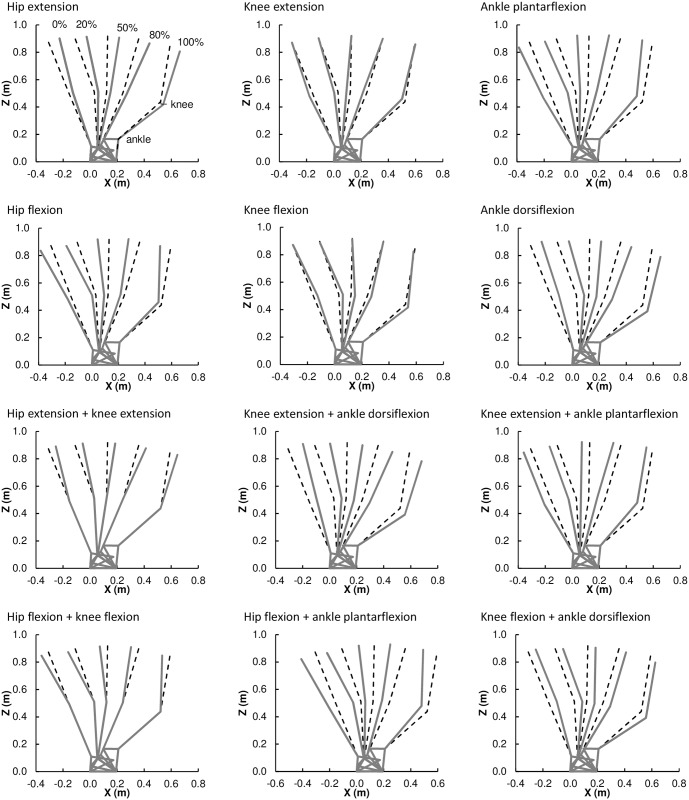
Diagram of lower limb postures after joint angle manipulation (gray) in comparison to the original posture (dashed black). The particular joint angle changes for each manipulation are specified in Table 3. Postures at 0%, 20%, 50%, 80%, and 100% of stance are depicted from left to right.

Joint angle manipulation was limited to the hip, knee, and ankle joints without affecting the position of the foot and pelvis (relatively to the ground). Although changes in the position of the foot and pelvis could presumably accompany changes in joints during human walking, keeping them constant in our model greatly narrows possible postural solutions. Given the above condition, any change in a joint angle has to be accompanied with a change in the other joint(s) angle following the relationship:

Δω=Δε+Δγ (3)

where ω, ε and γ are the flexion/extension angles at the hip, knee and ankle, respectively (Fig 4). We manipulated each joint by adding/subtracting 2SD (i.e., 8°) while the change in the other joint angles was not allowed to exceed the change in the manipulated joint (i.e., ≤ 8°). The solutions of the above conditions are summarized in Table 2. Fig 5 shows positions of the lower limb segments after the particular manipulations of joint angles in which either one joint is changed by 8°and the other two by 4° or two joints are changed by 8°and the remaining one is not changed.

**Table 2:** Postural changes at the hip, knee and ankle during the joint angle manipulation.

Joint angle manipulation	ΔHip angle (ω) (°)	ΔKnee angle (ε) (°)	ΔAnkle angle (γ) (°)
Hip extension	−8	[−8, 0]	[−8, 0]
Hip flexion	8	[0, 8]	[0, 8]
Knee extension	[−8, 0]	−8	[0, 8]
Knee flexion	[0, 8]	8	[−8, 0]
Ankle plantarflexion	[0, 8]	[−8, 0]	8
Ankle dorsiflexion	[−8, 0]	[0, 8]	−8

**Table 3:** Effect of 2 SD change of joint angles on peak net moments at the hip, knee and ankle evaluated as a change from the original value in N m and percentages.

Manipulated parameter	ΔJoint angle (°)			ΔPeak net joint moment (N m)											
				Hip				Knee				Ankle			
	Hip	Knee	Ankle	Flex		Ext		Flex		Ext		Plant		Dors	
Hip ext	−8	−4	−4	−12.5	(23%)	9.5	(12%)	−1.9	(10%)	1.8	(6%)	−2.5	(19%)	4.8	(5%)
Hip flex	+8	+4	+4	13.3	(25%)	−9.7	(12%)	1.9	(10%)	−1.9	(7%)	2.7	(20%)	−4.8	(5%)
Knee ext	−4	−8	+4	1.7	(3%)	0.3	(0%)	−18.6	(97%)	16.9	(60%)	0.0	(0%)	0.0	(0%)
Knee flex	+4	+8	−4	−2.2	(4%)	−0.8	(1%)	18.4	(96%)	−16.3	(58%)	0.0	(0%)	0.0	(0%)
Ankle plant	+4	−4	+8	15.5	(26%)	−8.9	(11%)	−16.7	(87%)	15.0	(52%)	2.7	(19%)	−4.8	(5%)
Ankle dors	−4	+4	−8	−14.2	(29%)	9.0	(11%)	16.6	(87%)	−14.5	(53%)	−2.6	(20%)	4.8	(5%)
Hip ext + knee ext	−8	−8	0	−7.3	(13%)	6.5	(8%)	−13.7	(71%)	12.4	(44%)	−1.7	(13%)	3.2	(3%)
Hip flex + knee flex	+8	+8	0	7.2	(13%)	−7.1	(9%)	13.6	(71%)	−12.2	(44%)	1.7	(13%)	−3.2	(4%)
Hip ext + ankle dors	−8	0	−8	−17.5	(32%)	12.5	(16%)	9.8	(51%)	−8.6	(31%)	−3.3	(25%)	6.5	(7%)
Hip flex + ankle plant	+8	0	+8	19.6	(36%)	−12.3	(15%)	−9.9	(52%)	8.6	(31%)	3.7	(27%)	−6.3	(7%)
Knee ext + ankle plant	0	−8	+8	11.4	(21%)	−5.7	(7%)	−23.5	(123%)	21.4	(76%)	1.8	(13%)	−3.2	(3%)
Knee flex + ankle dors	0	+8	−8	−11.1	(20%)	5.4	(7%)	23.3	(122%)	−20.4	(73%)	−1.8	(13%)	3.2	(3%)

In joint moment changes, positive values indicate increase of the peak and negative values indicate decrease of the peak; ext, extension; flex, flexion; plant, plantarflexion; dors, dorsiflexion; the resulting posture for each manipulation is shown in Fig 5.

#### Step 3: Inverse dynamics

Third, we calculated the net joint moments for the average individual (S1 File) and all the parameter manipulations. We calculated the coordinates of the lower limb joints and COMs of the lower limb segments throughout the stance phase with the origin of the coordinate system at the posterior margin of the foot projected on the ground at the heel strike. The net joint moments at the hip, knee, and ankle were calculated from these coordinates using the basic link-segment equations [55]:

$$\sum F_{x}=m\times a_{x}$$ (4)

$$\sum F_{y}=m\times a_{y}$$ (5)

$$\sum M=I_{0}\times \alpha$$ (6)

where ΣF_x_ is the sum of the reaction forces acting on the segment in the anteroposterior direction, ΣF_y_ is the sum of the reaction and gravitational forces acting on the segment in vertical direction, ΣM is the sum of the moments acting about the segment COM, m is the segment mass, a_i_ is the acceleration of the segment COM, I_0_ is the moment of inertia about the segment COM, and α is the angular acceleration of the segment. The equations were solved successively for the foot, shank, and thigh. For calculation of accelerations we assumed stance time to be 0.661 s, which was the mean stance time in our human sample. The relative radius of gyration and relative position of COM of the lower limb segments and head-arms-trunk segment were taken from Winter [55].

To account for a possible source of error in our net joint moment estimates associated with the uncertainty of the DP location after the size and postural manipulations (Gruben and Boehm [64] reported SD of their mean DP vertical location to be 0.13 of COM_body_ height) we calculated the net joint moments for all the size and postural manipulations also with the DP located at 0.8 and 0.26 of the COM_body_ height above the hip, i.e., at the previously reported mean ± 2SD DP locations.

#### Accuracy of the DP model

The accuracy of the DP model was assessed experimentally in a sample of ten non-obese adults (4 males, 6 females; age: 29.8 ±7.5 years; body mass: 68.3 ± 17.8 kg, stature: 1.676 ± 0.116 m). Each participant provided written informed consent prior to participation, and the protocol was approved by the University Integrated Institutional Review Board, Hunter College, City University of New York. The participants walked at their preferred speed on a walkway while their complete ground reaction force data were recorded by a force plate (AMTI, Watertown, USA) at 1000 Hz, synchronously with marker trajectories (whole body Plug-In Gait marker set) by a 6-camera motion capture system (Vicon, Oxford, UK) at 200 Hz. The marker trajectories, ground reaction force, and center of pressure were filtered using a fourth-order low-pass Butterworth filter with a 6-Hz cut-off frequency [55,81,82]. Net joint moments were calculated using Eqs 4–6. The height of the COM_body_ at the standing posture was estimated using the same approach as in our average individual model following Winter [55]. The accuracy of the DP model in estimation of the GRF_ap_ and net joint moments was assessed by mean error (ME), percentage mean error (%ME), mean absolute error (MAE), and percentage mean absolute error (%MAE) as follows:

$$ME=\frac{\sum_{i=1}^{n}\left(predicted_{i}-observed_{i}\right)}{n}$$ (7)

$$\%ME=\frac{\sum_{i=1}^{n}\left[\left(predicted_{i}-observed_{i}\right)/observedrange_{i}\right]\times 100}{n}$$ (8)

$$MAE=\frac{\sum_{i=1}^{n}\left|\left(predicted_{i}-observed_{i}\right)\right|}{n}$$ (9)

$$\%MAE=\frac{\sum_{i=1}^{n}\left[\left|\left(predicted_{i}-observed_{i}\right)\right|/observedrange_{i}\right]\times 100}{n}$$ (10)

where predicted_i_ is the estimated parameter value in the i^th^ individual, observed_i_ is the parameter value calculated from the complete GRF data, observedrange_i_ is the range of the variable within stance phase calculated from the complete GRF data (e.g., difference between flexion and extension peak of the net hip moment calculated from the complete GRF data), and n is the sample size [83]. The individual net joint moment range was used in computation of %ME and %MAE since results of previous studies [84,85] suggest that the joint moment ranges are more consistent between different kinematic models (we used PiG model for DP accuracy test and CAST model for the main data collection) than the peak joint moments. For GRF_ap_, the range was used for consistency of the %ME and %MAE computation.

The ME, %ME, MAE, and %MAE of the parameter estimates are presented in Table 4. The DP model consistently underestimates the GRF_ap_ peaks and the peak knee flexion moment, overestimates the peak knee extension moment and the hip moment peaks, and provides excellently accurate estimate of the ankle moment peaks.

**Table 4:** Errors of estimates of the peak GRF_ap_ and peak net joint moments (mean (SD)).

Parameter	Peak	Mean error	%Mean error (%)	Mean absolute error	%Mean absolute error (%)
GRF_ap_ (N)	posterior	18.0 (15.0)	7.1	18.9 (13.7)	7.8
	anterior	−15.9 (13.1)	−6.3	15.9 (13.1)	6.3
Ankle moment (N m)	plantarflexion^a^	−0.9 (0.8)	−0.8	1.0 (0.6)	0.9
	dorsiflexion	0.4 (1.0)	0.5	0.8 (0.6)	0.9
Knee moment (N m)	flexion	−7.2 (5.9)	−18.0	7.4 (5.7)	18.3
	extension^b^	−4.7 (3.9)	−17.7	4.9 (3.7)	18.0
Hip moment (N m)	flexion	5.6 (8.0)	5.8	7.9 (5.5)	8.9
	extension	−11.9 (10.2)	−13.2	12.1 (9.8)	13.5

^a^sample size is reduced to n = 8 because ankle plantarflexion moment was present only in eight subjects

^b^sample size is reduced to n = 7 because knee extension moment was present only in seven subjects

### Analysis of experimental data

To evaluate the independent impact of body mass and lower limb length on lower limb posture during walking (our second goal) we analyzed the experimental data using general linear model (Fig 1).

#### Step 4: General linear model

The relationship between body size (body mass and lower limb length) and posture (joint flexion angles) was analyzed at events of expected peak net joint moments. Six gait events (two per each joint) were selected to track the expected peak net joint moments (Fig 3): the first peak of the vertical GRF to track the peak net hip flexion moment (H-flex), maximal hip extension to track the peak net hip extension moment (H-ext), maximal knee flexion at the first half of stance to track the peak net knee flexion moment (K-flex), maximal knee extension at the second half of stance to track the peak net knee extension moment (K-ext), maximal ankle plantarflexion at the first half of stance to track the peak net ankle plantarflexion moment (A-plant), and the second peak of the vertical GRF to track the peak net ankle dorsiflexion moment (A-dors). Relationships between variables were analyzed using Pearson product-moment correlation coefficients. Because velocity is positively correlated with the hip and knee flexion angle at early stance events, our attempt to control for velocity using preferred velocity was not successful. Velocity was thus inserted into our general linear model analyses as an extra variable to enable controlling for its effect. The general linear model with type VI sums of squares and commonality analysis was used to evaluate the independent influence of each body size variable on joint flexion while controlling for the other size variable, sex, and velocity. Commonality analysis [86,87] (see the recent applications and examples in [88]) was used to determine the unique effect of each variable in the model and the common effect of the body size variables. Statistical analyses were performed using Statistica 10 (StatSoft, Tulsa, OK, USA) and Excel 2013 (Microsoft, Redmond, WA, USA).

## Results

### Effect of body size on net joint moments

**Fig 6:**
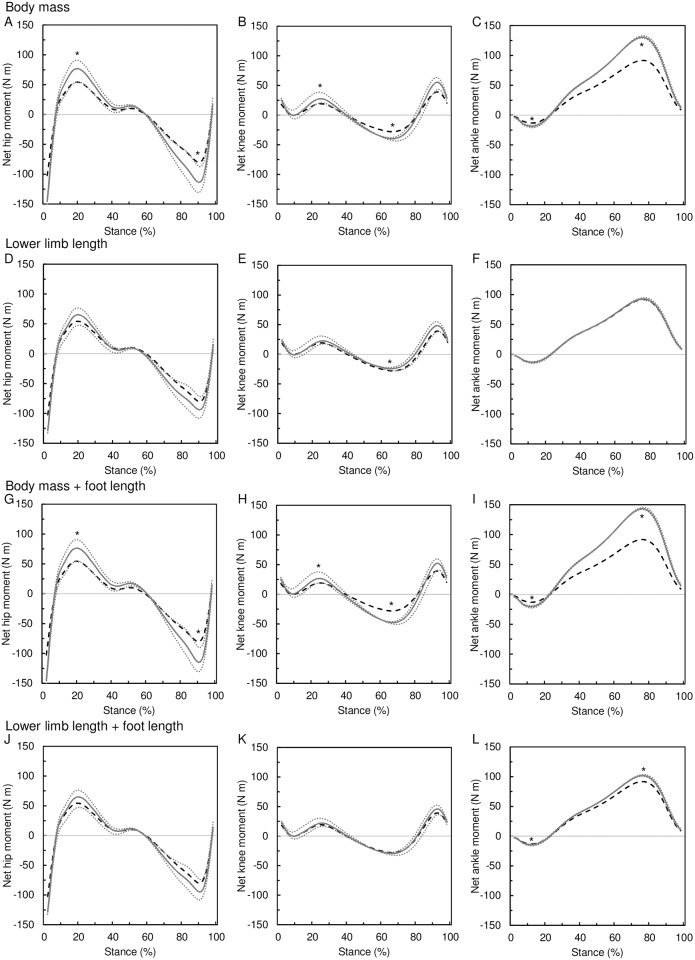
Effect of body size on net joint moments. The effect of body mass increase (A–C) and lower limb length increase (D–F) on net joint moments at the hip (A, D), knee (B, E), and ankle (C, F). Manipulated size (gray solid line) is compared to the original size (black dashed line). The gray dotted lines represent the moments after the size manipulation estimated using the mean ± 2 SDs divergent point location. The asterisk indicates the peak joint moment of the manipulated body size is different from the original moment regardless of the divergent point location.

The effect of 2 SD increments of body size on peak net joint moments is presented in Table 5 and Fig 6. Body mass has a positive, almost directly proportional effect on all peak net moments at all three joints: a 43% (= 2 SD) increase in body mass leads to 41–43% increase in the peak joint moments. The effect of lower limb length on peak net joint moments is only roughly proportional at the hip and knee but disproportionally low at the ankle. A 15% (= 2 SD) increase in lower limb length increases the peak hip moments by 17–20%, peak knee flexion moment by 18% and peak ankle moments by 1–2%, whereas it decreases the knee extension moment by 13%. Thus, the lower limb length *per se* has a positive effect on net moments at the hip and ankle and on the knee flexing moment, but a negative effect on the knee extension moment at late stance. The inclusion of the foot length prolongation in body size manipulations has a little effect on the resulting peak hip moments (about 1%) and knee flexion moments (4%), but it increases the magnitude of the ankle moments by 14% and 9% in body mass and lower limb manipulation respectively. Nevertheless, the most pronounced effect of foot length prolongation is on knee extension moment that increases by 28% and 18% in body mass and lower limb manipulation, respectively.

**Table 5:** Effect of 2 SD increase of body mass and lower limb length on peak net moments at the hip, knee and ankle evaluated as a change from the mean value in N m and percentages.

Manipulated parameter	ΔParameter	ΔPeak net joint moment (N m)											
		Hip				Knee				Ankle			
		Flex		Ext		Flex		Ext		Plant		Dors	
Body mass	+29 kg (41%)	22.5	(41%)	33.6	(42%)	8.3	(43%)	12.0	(43%)	5.5	(41%)	38.8	(42%)
Lower limb length	+126 mm (15%)	10.9	(20%)	13.7	(17%)	3.4	(18%)	−3.8	(13%)	0.2	(2%)	1.1	(1%)
Body mass + foot length	+29 kg (41%); +15 mm (6%)	21.9	(40%)	34.4	(43%)	7.4	(39%)	19.8	(71%)	7.3	(55%)	51.7	(56%)
Lower limb length + foot length	+126 mm (15%); +18 mm (7%)	10.6	(19%)	14.7	(18%)	2.6	(14%)	1.5	(5%)	1.5	(11%)	10.0	(11%)

Notes: positive values indicate increase of the peak moment, negative values indicate decrease of the peak moment; flex, flexion; ext, extension; plant, plantarflexion; dors, dorsiflexion.

### Effect of posture on net joint moments

**Fig 7:**
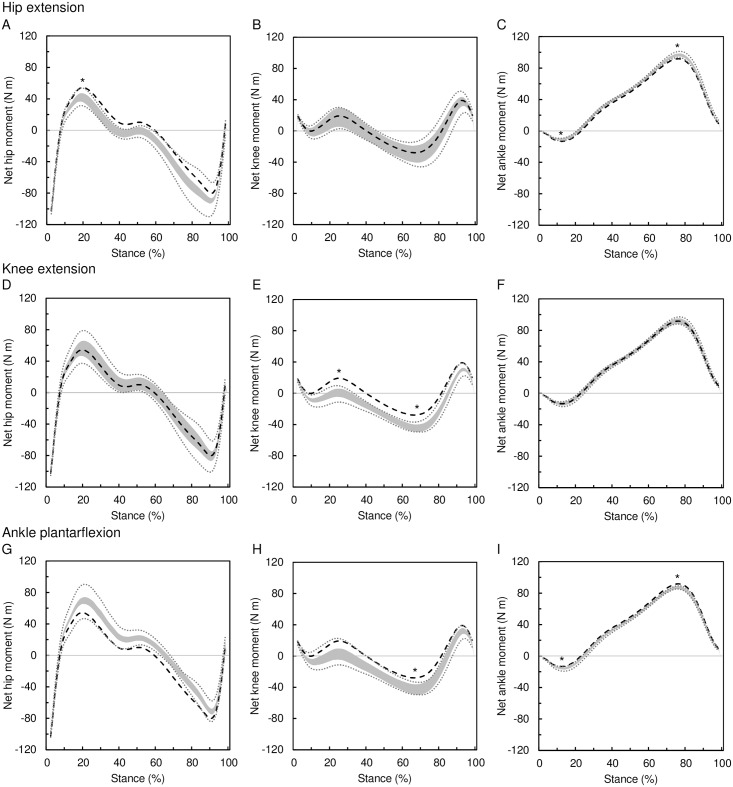
Effect of posture on net joint moments. The effect of hip extension (A–C), knee extension (D–F), and ankle plantarflexion (G–I) on net joint moments at the hip (A, D, G), knee (B, E, H), and ankle (C, F, I). Manipulated postures (gray area) are compared to the original posture (black dashed line). The gray area covers all possible postural changes meeting the following conditions: 1) the manipulated joint change is 8°, 2) the change in other joints is ≤ 8°, 3) Δhip angle = Δknee angle + Δankle angle. See the text for details and Table 2 for intervals of angle change in other joints meeting the three conditions. The gray dotted lines represent the range of moments after posture manipulations estimated using the mean ± 2 SDs divergent point location. The asterisk indicates the peak moment of the manipulated posture is different from the original moment regardless of the divergent point location.

The effect of posture on net joint moments at the hip, knee, and ankle is shown in Table 3 and Fig 7. The greatest postural effect on net joint moment was observed at the knee (up to 23.5 N m) followed by the hip (up to 19.6 N m) and ankle (under 6.5 N m). The relative effect at the knee (up to 123%) is 3.5 times greater than that at the hip (up to 36%) and 4.5 greater than that at the ankle (up to 27%). Hip extension results in the reduction of the hip flexion moment (13–32%) and ankle plantarflexion moment (13–25%), whereas it increases the hip extension moment (8–16%) and ankle dorsiflexion moment (3–7%). The knee extension results in the reduction of the knee flexion moment (71–123%) and increase of the knee extension moment (44–76%). The ankle plantarflexion reduces the hip extension moment (7–15%), knee flexion moment (52–123%), and ankle dorsiflexion moment (3–7%), whereas it increases the hip flexion moment (21–36%), knee extension moment (31–76%), and ankle plantar flexion moment (13–27%). Thus, the knee joint moment can be most effectively affected by postural changes, particularly by the changes of the knee angle.

### Body size and joint flexion at gait events

Table 6 shows the pairwise Pearson’s correlation coefficients of the body size parameters, velocity, and flexion angles at the hip and knee at selected gait events. Body mass is positively correlated with lower limb length (r = 0.7). Furthermore, body mass is negatively correlated with the knee flexion angle at both analyzed events (r = −0.3) and with ankle dorsiflexion angle (r = −0.3), whereas lower limb length is correlated only with velocity (r = 0.3) and not with any joint angle.

**Table 6:** Pearson product-moment correlation coefficients between body size parameters, velocity and flexion angles at selected gait events in a pooled sex sample (n = 49).

	Lower limb length	Velocity	Hip flexion angle		Knee flexion angle		Ankle flexion angle	
			H-flex	H-ext	K-flex	K-ext	A-plant	A-dors
**Body mass**	0.714	0.220	−0.178	0.089	−0.297	−0.285	0.160	0.337
**Lower limb length**		0.326	−0.142	−0.099	−0.055	−0.189	–0.0003	0.234
**Velocity**			0.595	−0.087	0.426	0.198	–0.011	0.152

Gait events: H-flex, peak hip flexion moment; H-ext, peak hip extension moment; K-flex, peak knee flexion moment; K-ext, peak knee extension moment; A-plant, peak ankle plantarflexion moment; A-dors, peak ankle dorsiflexion moment.

The results of the general linear model and commonality analysis are presented in Table 7. The regression model, which includes body mass, lower limb length, velocity, and sex, explains 51.5% of the variation in the hip flexion angle at the peak hip flexion moment (H-flex) and 43.8% of the variation in the knee flexion angle at the peak knee flexion moment (K-flex). The model is not significant at later stance events, peak hip extension moment (H-ext), and peak knee extension moment (K-ext), and at the ankle events. Body mass has a significant negative effect on knee angle at K-flex (p = 0.001), whereas lower limb length has a significantly negative effect on hip angle at H-flex (p = 0.033). Body mass uniquely explains 15.8% of the variance in knee flexion at K-flex. Lower limb length uniquely explains 5.4% of the variance in hip flexion at H-flex. In addition to the uniquely explained variance, body mass and lower limb length share 9% of the explained variance at both events. Thus, the body size variables together account for 16% and 25% of the variance in hip flexion at H-flex and knee flexion at K-flex, respectively. Nevertheless, velocity is the main determinant of joint angle at both these events, uniquely explaining 43.2% and 18.6% of the joint angle variance at H-flex and K-flex, respectively. In addition to body mass and velocity, the knee flexion angle at K-flex is also affected by sex (uniquely explaining 8.7% of variance). At K-flex, females tend to keep the knee more extended than do males.

**Table 7:** Effect of body mass, lower limb length, velocity, and sex on joint flexion angles at selected gait events using general linear models.

Joint angle	Event	Model R^2^	Variable^a^	b	U	C_B,L_	U_B_+U_L_+C_B,L_
Hip	H-flex	0.515***	Body mass	−0.069	0.014	0.089	0.156
			Limb length	−0.034*	0.054		
			Velocity	7.865***	0.432		
			Sex	1.156	0.026		
	H-ext	0.066					
Knee	K-flex	0.438***	Body mass	−0.239**	0.158	0.091	0.249
			Limb length	−3.4×10^−4^	<0.001		
			Velocity	5.290***	0.186		
			Sex	2.179*	0.087		
	K-ext	0.185					
Ankle	A-plant	0.151					
	A-dors	0.149					

b, slope adjusted for effect of all other variables in the model; U, proportion of unique variance explained by variable; C_B,L_, proportion of common variance shared by body mass and lower limb length; U_B_+U_L_+C_B,L_, proportion of variance explained cumulatively by the body mass and lower limb length; H-flex, peak hip flexion moment; H-ext, peak hip extension moment; K-flex, peak knee flexion moment; K-ext, peak knee extension moment; A-plant, peak ankle plantarflexion moment; A-dors, peak ankle dorsiflexion moment;

* p < 0.05;

** p < 0.01;

*** p < 0.001.

^a^the effect of particular variables is shown only for models that explain significant portion of variation in joint flexion angle.

## Discussion

Our model demonstrates that the size-related increase of net moments at the knee can be effectively compensated by relatively small postural adjustments, especially by changes of the knee flexion angle. The hip flexion moment and ankle plantarflexion moment can also be, to some degree, moderated by postural adjustments, but the hip extension moment and ankle dorsiflexion moment are relatively resistant to the changes of posture within the variation in normal walking adults. The changes of the knee flexion angle have far the greatest effect on net joint moments in comparison to the changes of the angle at the hip and ankle. Moreover, the moderation of the particular joint moment by changing the ankle posture has a side effect—simultaneous increase of the net moment at the other joint(s) (e.g., ankle plantarflexion moderates the knee flexion moment but increases the hip flexion moment and ankle plantarflexion moment; Fig 7G–7I). The moderation of the knee moments by knee angle adjustment has no such side effect since the changes of the knee flexion have little effect on the other joint moments. The hip joint angle adjustment can result in simultaneous moderation of the hip and ankle moments; however, its effect is far subtler than that of the knee. Based on the results of our model, it can be predicted that body mass-related and/or lower limb length-related increase of knee flexion moment at early stance would be most effectively compensated by knee extension, whereas the body mass-related increase of the knee extension moment at late stance could be compensated by knee flexion. No change of posture at late stance is expected to be associated with prolongation of the lower limb, since our model shows that the knee extension moment at late stance actually decreases with lower limb prolongation.

Previous experimental studies [64,79] reported that the GRF vector consistently intersects near the DP located above the hip joint during walking in humans. This observation was supported by strong coefficient of determination (r ^2^ = 0.996) between the σ angle (the angle between the GRF vector and the horizontal) calculated from complete force plate data and σ angle estimated by the DP model [64]. Our test of the DP model accuracy (Table 4) shows that the DP model provides accurate estimates of the peak joint moments at the hip, knee, and ankle, with errors <20% for flexion and extension across all three joints and <1% for the ankle. The largest errors were evident at the knee (~18% in both flexion and extension) and hip extension (~13%). The similar magnitude of ME and MAE values (Table 4) indicates that the DP model consistently underestimated (knee flexion) or overestimated (knee extension and hip extension) these moments. We were able to lower the error at these moments below 10% by moving the DP location inferiorly (by 11.5% of the COM_body_ height) and posteriorly (by 0.8% of the COM_body_ height), which suggests that the mean DP may be shifted in our sample in comparison to the previous study [64]. Nevertheless, the %ME and %MAE of these peak moments are not correlated with body mass, lower limb length, and corresponding peak flexion/extension angle. Since this demonstrates that the accuracy of the model is unrelated to the parameters tested in this study, the detected errors cannot substantively affect the pattern of our results.

There are no data available at the moment to infer whether the relative vertical position of the DP is unaffected by body mass, lower limb length or posture as is presumed in our model. To account for this uncertainty we estimated the joint moment also with DP located at the previously reported mean±2SD above the hip and present the results in Figs 6 and 7. In Fig 6 we show that body mass increases the peak net moments at all the three joints regardless of the relative DP location. This is true also when foot length is manipulated along with body mass. The effect of lower limb length on hip moment peaks and knee flexion moment peak is, however, affected by the relative DP location. If the prolongation of lower limb is associated with the shift of the DP location closer to the hip, then the peak moments at the hip would not be increased. On the other hand, a possible association of the limb prolongation with DP shift further from the hip would limit the increase of the peak knee flexion moment. Fig 7 shows the scenario when the posture is associated with the relative DP location. The effect of the hip extension on peak joint moments is not affected by relative DP location with the exception of the hip extension moment. The effect of knee flexion on peak joint moments is not affected by relative DP location. The effect of ankle plantarflexion on peak hip moments and peak knee flexion moment is, however, affected by the relative DP location; although the relative DP location would actually turn the effect to the opposite direction only for few postural combinations. We conclude that our model predictions are relatively robust over the biologically feasible range of variation in relative DP location.

In our model manipulations, stance time was assumed to be unrelated to changes in body size parameters. Examination of this assumption by additional general linear model analysis of our experimental data revealed that body mass is not correlated with stance time after controlling for lower limb length, velocity, and sex. This finding corresponds well to previous studies reporting nonsignificant difference in stance time between lean and obese subjects [89,90]. Nevertheless, we detected significant although weak correlation between stance time and lower limb length after controlling for body mass, velocity, and sex (p = 0.011; b = 0.00029 s/mm; lower limb length uniquely explains 7.4% of variance in stance time). Thus, we simulated the effect of simultaneous increase of lower limb length and stance time on net joint moments using the slope from the general linear model. The results show that appropriate increase of stance time slightly reduces the effect of lower limb length on net hip moments (by 5–6%) and knee joint moments (by 1–2%). No apparent effect has been detected at the ankle (under 0.5%).

Our experimental results agree with some of our model predictions. Particularly, we detected that body mass is negatively correlated with flexion at the knee at early stance. Using our model, this finding can be interpreted as a strategy to moderate the body mass-related increase of knee flexion moment by knee extension and it also corresponds to the previous findings in humans [32–34] and other mammals [2,27–29]. On the other hand, we did not detect any body mass-related postural adjustments at late stance. In addition, we detected a significant negative correlation between the lower limb length and flexion at the hip at early stance. Although this finding can be also interpreted as a strategy to moderate the increase of hip flexion moment, we point out that the correlation is weak and the lower limb length uniquely explains only 5.4% of the variance in hip flexion angle.

The moment-moderation efficiency of the detected postural adjustments can be roughly assessed using our model as a basis. According to our model, the peak knee flexion moment at early stance increases by 0.29 N m per kilogram of body mass (i.e., the effect of 2 SD change of body mass on peak knee flexion moment divided by 2 SD of body mass) and decreases by 1.71–2.94 N m per degree of knee extension. The peak flexion moment at the hip increases by 0.87 N m per centimeter of lower limb length and decreases by 0.46–1.10 N m per degree of hip extension. Thus, to cancel the size-related increase of moments, knee extension would need to increase at the rate 0.10–0.17° kg^–1^ and hip extension at the rate 0.79–1.89° cm^–1^. The 0.24° kg^–1^ effect of body mass on knee extension detected in our sample (Table 7) is well above the estimated minimum rates, which indicates that the detected postural adjustments at the knee are adequate for complete cancellation of the body mass-related knee moment increase. The 0.34° cm^–1^ effect of lower limb length on hip extension detected in our sample is, however, lower than the estimated minimum rate, which suggests that the limb length-related increase of hip moment would be only moderated but not cancelled out by the detected hip angle adjustment.

The presumed complete cancellation of the body mass-related increase of knee flexion moment corresponds well to the results of Gushue et al. [34] who found that obese children walk with reduced knee flexion at early stance and with no significant differences in the peak knee flexion moments when compared to lean children. However, in the comparison of obese and lean adult kinematics, DeVita and Hortobágyi [33] detected differences in all three lower limb joints. Particularly, the obese walked with reduced flexion at the hip and knee and greater plantarflexion at the ankle at early stance. In our study, however, only the knee flexion angle is significantly related to body mass and the hip and ankle angles are not. A possible explanation could be that reduced hip flexion at early stance not only moderates hip moments, but also shortens the step, which would negatively affect the velocity and increase the number of steps to cover a given distance. In the sample of DeVita and Hortobágyi [33], the body mass difference was likely so great that the joint moment moderation effect of hip extension outweighed the shortening of the step length. Our sample, however, consists of non-obese individuals among whom body mass may not be such a limiting factor as to compromise step length. Thus, in our sample, only longer-limbed individuals can afford to moderate hip moment by step-shortening changes of posture, whereas individuals of greater body mass moderate only the knee moment. Nevertheless, it is not clear why longer limbed individuals do not moderate moment at the knee at early stance.

Although the size-related postural adjustments at the hip and knee moderate the size-related increase of net joint moments according to our model, the variation in flexion at the hip and knee explained by body size parameters is not particularly strong. We detected that part of the variation in hip and knee flexion is accounted for by the effect of velocity, and also by sex in the case of knee flexion, which corresponds with previous studies [43–53]. The weakness of the relationship, the sex effect, and the persistence of the velocity effect even when walking at preferred speed likely prevented the detection of the body mass-knee flexion relationship at early stance in previous studies [32]. Other factors such as individual body mass history and lower limb musculoskeletal strength could also be of importance. Additionally, the loads imposed on hip and knee joints by walking may not be sufficient to more closely relate body size to posture. Our results do not support the conclusions of Gruss [35] that longer limbed individuals keep more extended knees at late stance to moderate knee moments. Our model predicts that lower limb length has a negative effect on knee extending moment at late stance. Thus, the knee extending moment at late stance is actually lower in longer-limbed individuals and as such does not need to be moderated. Moreover, our model shows that the knee extending moments at late stance cannot be moderated by greater knee extension but by greater knee flexion. Nevertheless, it would be expected that individuals of greater body mass would moderate the knee moment at late stance because it increases with body mass. Such negative correlation between body mass and knee flexion at late stance was even previously reported in humans [32]. Despite that we also detected elevated negative correlation between body mass and knee flexion in late stance, the relationship was not significant when lower limb length, velocity and sex were controlled for in the general linear model. We hypothesize that the knee extending moment at late stance could be beneficial for stabilizing the knee against the action of the gastrocnemius muscle, which is contracting vigorously to counteract the dorsiflexion moment at the ankle during the second half of stance [91].

The results of the present study could be of importance for clinicians as the body size should be considered when analyzing deviations from normal gait patterns. Also, future simulations of past human locomotion based on kinematics of recent humans should take into account the link between body size and posture detected here. In the present study, we analyzed the effect of body size and posture on net joint moments, which only reflects the net effect of agonist and antagonist muscles. Nevertheless, several combinations of muscle forces can produce the same net joint moment and also the passive structures such as the ligaments can contribute to the net joint moment [55,58]. Future studies may build on the present work and use the advantages of musculoskeletal modeling [10,92–95] to analyze the effect of the postural changes on the muscle force and consequently the locomotor costs [7,9,96,97] and loading of the bones [98–101]. Also, complex gait modeling approaches [95] may explore the function of the detected relationship between body size and locomotor posture by determining which optimization parameters fit best the relationship detected here.

## Conclusions

In the present study, we detected that body size is negatively related to flexion at the hip and knee at early stance of walking in non-obese humans. Body mass is negatively related to the knee flexion, whereas lower limb length is negatively related to the hip flexion. According to our model, the detected postural adjustments are sufficient to cancel the size-related increase of the knee moment and moderate the increase of the hip moment. The variance in flexion at the hip and knee explained by body size parameters together is less than 25%. Velocity has the greatest effect on flexion at both the hip and knee, whereas sex is also a significant factor at the knee. The relatively weak association of body size with posture could be a consequence of the relatively low mechanical loading of lower limbs during the walking gait.

## Supporting information

S1 Table Subject anthropometric and gait parameters. (DOCX)

S1 File Model for net joint moments estimation. (XLSX)
