# Supplementary material for: Body size and lower limb posture during walking in humans
Source: PLoS One. 2017 Feb 13;12(2):e0172112. doi: 10.1371/journal.pone.0172112 (PMC5305206; doi:10.1371/journal.pone.0172112)
Supplement: S1 Table — (DOCX) [file pone.0172112.s001.docx]

**S1 Table. Subject anthropometric and gait parameters**

| Ind. # | Sex | Body mass (kg) | Lower limb length (mm) | Velocity (m s^−1^) | Hip flexion angle at H-flex (°) | Hip flexion angle at H-ext  (°) | Knee flexion angle at K-flex (°) | Knee flexion angle at K-ext  (°) | Ankle flexion angle at A-plant (°) | Ankle flexion angle at A-dors  (°) |
| --- | --- | --- | --- | --- | --- | --- | --- | --- | --- | --- |
| 1 | M | 67.2 | 811 | 5.1 | 18.1 | −20.1 | 20.6 | 5.7 | 4.8 | −17.4 |
| 2 | F | 74.6 | 779 | 4.0 | 9.0 | −16.6 | 8.6 | −1.0 | 8.1 | −8.6 |
| 3 | M | 79.7 | 840 | 4.4 | 13.9 | −17.5 | 13.4 | 0.1 | 4.3 | −10.0 |
| 4 | M | 73.2 | 897 | 4.6 | 12.5 | −16.9 | 12.6 | 2.1 | 4.6 | −13.6 |
| 5 | F | 53.1 | 742 | 3.5 | 7.1 | −18.1 | 8.4 | 3.7 | 6.4 | −12.3 |
| 6 | M | 72.4 | 859 | 4.8 | 12.9 | −18.7 | 18.1 | −2.8 | 5.2 | −3.1 |
| 7 | F | 62.1 | 822 | 3.9 | 4.7 | −22.7 | 15.4 | −2.2 | 3.9 | −13.3 |
| 8 | M | 62.8 | 785 | 4.7 | 17.3 | −19.7 | 24.8 | 7.3 | −0.2 | −13.9 |
| 9 | M | 97.1 | 950 | 4.9 | 8.7 | −16.8 | 8.1 | 1.6 | 6.0 | −11.5 |
| 10 | M | 53.2 | 746 | 4.9 | 14.6 | −21.9 | 20.7 | −0.8 | 2.7 | −7.3 |
| 11 | M | 78.1 | 842 | 5.2 | 10.4 | −12.4 | 9.8 | 3.3 | 3.3 | −11.0 |
| 12 | M | 84.3 | 882 | 5.3 | 17.6 | −15.9 | 21.9 | 2.4 | 5.1 | −9.3 |
| 13 | M | 95.5 | 842 | 5.0 | 18.6 | −15.3 | 19.9 | 1.0 | 4.8 | −7.7 |
| 14 | M | 91.0 | 976 | 5.6 | 8.1 | −23.2 | 14.5 | −3.0 | 7.0 | −6.0 |
| 15 | M | 72.0 | 856 | 4.9 | 15.0 | −19.2 | 17.9 | 2.1 | 1.9 | −8.7 |
| 16 | F | 52.7 | 766 | 4.3 | 10.4 | −16.8 | 7.2 | −3.0 | 9.5 | −13.3 |
| 17 | F | 77.7 | 816 | 4.6 | 11.4 | −19.6 | 16.3 | −1.4 | 5.1 | −10.7 |
| 18 | F | 63.0 | 818 | 4.8 | 6.5 | −19.3 | 20.7 | 4.6 | 4.3 | −13.0 |
| 19 | M | 86.5 | 898 | 5.1 | 11.5 | −18.6 | 14.0 | −0.6 | 5.2 | −9.7 |
| 20 | F | 61.0 | 802 | 6.0 | 19.7 | −27.4 | 18.3 | 1.1 | 7.1 | −12.1 |
| 21 | F | 64.9 | 820 | 5.0 | 10.0 | −12.8 | 9.6 | 0.6 | 7.9 | −8.6 |
| 22 | M | 67.0 | 856 | 5.0 | 15.4 | −17.5 | 22.9 | 4.9 | 3.0 | −13.4 |
| 23 | M | 90.0 | 870 | 5.4 | 12.9 | −17.3 | 20.2 | 10.4 | 4.7 | −12.4 |
| 24 | F | 36.3 | 723 | 3.8 | 11.7 | −14.7 | 13.6 | 4.7 | 5.2 | −13.8 |
| 25 | M | 85.5 | 791 | 5.3 | 23.7 | −13.8 | 16.6 | 0.4 | 6.5 | −11.8 |
| 26 | M | 60.1 | 816 | 5.7 | 21.8 | −16.6 | 18.8 | 8.8 | 3.5 | −11.6 |
| 27 | F | 54.5 | 704 | 5.4 | 18.6 | −16.6 | 13.1 | 5.5 | 10.1 | −13.4 |
| 28 | F | 77.8 | 789 | 5.5 | 18.6 | −15.8 | 15.8 | −0.9 | 10.6 | −13.7 |
| 29 | F | 76.5 | 855 | 5.6 | 21.5 | −13.0 | 14.3 | 1.5 | 7.2 | −11.5 |
| 30 | F | 71.1 | 883 | 4.8 | 14.2 | −18.9 | 10.2 | −5.1 | 4.7 | −12.0 |
| 31 | M | 77.6 | 902 | 5.4 | 20.7 | −24.1 | 19.8 | −5.0 | 9.9 | −8.3 |
| 32 | M | 58.1 | 827 | 4.8 | 23.4 | −10.7 | 25.0 | 4.0 | 4.4 | −7.3 |
| 33 | F | 44.7 | 788 | 5.4 | 21.3 | −20.1 | 26.9 | 5.0 | 0.5 | −11.7 |
| 34 | F | 76.7 | 878 | 5.2 | 20.3 | −16.3 | 21.1 | 7.8 | 7.0 | −14.7 |
| 35 | M | 88.0 | 906 | 5.3 | 21.4 | −11.5 | 21.1 | 8.4 | 0.8 | −9.8 |
| 36 | M | 55.7 | 922 | 5.7 | 19.4 | −18.8 | 30.3 | 10.1 | 1.0 | −16.4 |
| 37 | M | 84.5 | 857 | 5.1 | 20.9 | −17.2 | 20.3 | 1.6 | 1.5 | −8.1 |
| 38 | F | 50.1 | 706 | 5.5 | 25.7 | −14.7 | 24.9 | 10.7 | 1.3 | −10.8 |
| 39 | F | 72.1 | 757 | 4.2 | 4.9 | −13.9 | 5.7 | 1.4 | 2.7 | −15.0 |
| 40 | M | 82.6 | 870 | 5.5 | 19.4 | −20.2 | 16.9 | −2.7 | 4.9 | −2.5 |
| 41 | F | 47.5 | 784 | 5.0 | 17.6 | −16.9 | 17.3 | 4.0 | 4.6 | −12.8 |
| 42 | F | 69.6 | 855 | 5.1 | 22.5 | −9.2 | 22.2 | 4.9 | 0.4 | −15.4 |
| 43 | F | 71.5 | 795 | 5.1 | 11.3 | −19.0 | 14.6 | −1.0 | 3.7 | −6.5 |
| 44 | M | 67.7 | 876 | 5.2 | 11.1 | −27.3 | 18.7 | 0.5 | 6.8 | −11.0 |
| 45 | F | 45.0 | 734 | 4.8 | 17.4 | −22.6 | 25.0 | 5.1 | 4.1 | −14.2 |
| 46 | F | 58.7 | 770 | 4.9 | 21.0 | −25.0 | 21.7 | −0.2 | 7.0 | −11.3 |
| 47 | M | 57.2 | 818 | 5.5 | 26.9 | −17.7 | 27.0 | 1.1 | 3.0 | −10.2 |
| 48 | F | 66.0 | 822 | 5.5 | 21.7 | −17.8 | 15.0 | 1.4 | 5.6 | −6.5 |
| 49 | F | 44.5 | 703 | 4.9 | 27.9 | −12.1 | 31.9 | 5.9 | 1.6 | −12.2 |

See text for parameters definitions.
